# Head impacts in Canadian varsity football: an exploratory study

**DOI:** 10.2217/cnc-2020-0024

**Published:** 2021-07-09

**Authors:** Laurie-Ann Corbin-Berrigan, Éric Wagnac, Sophie-Andrée Vinet, Camille Charlebois-Plante, Samuel Guay, Louis De Beaumont

**Affiliations:** 1 Département des sciences de l'activité physique, Université du Québec à Trois-Rivières, Trois-Rivières, QC, G9A 5H7, Canada; 2 Centre de recherche du CIUSSS du Nord-de-l'île de Montréal, Hôpital du Sacré-Coeur-de-Montréal, Montréal, QC, H4J 1C5, Canada; 3 Département de génie mécanique, École de Technologie Supérieure, Montréal, QC, H3C 1K3, Canada; 4 Département de psychologie, Université de Montréal, Montréal, QC, H2V 2S9, Canada; 5 Département de chirurgie, Faculté de médecine, Université de Montréal, Montréal, QC, H3C 3J7, Canada

**Keywords:** American football, concussion, head impact kinematics, head impacts, instrumented helmets, subconcussive head impacts

It is estimated that hundreds of thousands of Canadians of all ages play American football at all levels, through various organizations [1]. Although the positive health effects of organized sports such as football are well known and reported [2], health issues associated with contact sports have received a lot of attention due to concussion incidence [3]. In fact, when it comes to concussion incidence among organized sports, football is at the top of the list [4]. It is estimated that 85% of Canadian university football players will have sustained at least one concussion in their sporting career [5]. In recent years, the scientific community has raised the concern that practicing this contact sport could lead to long-term sequelae on brain function, such as chronic traumatic encephalopathy (CTE), a neurodegenerative disease of the brain, studied postmortem in a variety of contact sports players [6,7]. CTE research has led scientists to look above and beyond clinical history of concussion and to study head impacts from a broader perspective. A handful of researchers have linked head impact exposure, regardless of concussion occurrence, to alterations in cerebral structure and function in a variety of sports [8–12]. Many scientists agree that repeated head impacts could play a causative role for long-term deficits and neurodenegeration studied in retired athletes [13,14], such as CTE [9,10].

Head impacts happen when the head undergoes a sudden change in speed (acceleration or deceleration, linear or rotational), such as when colliding with an opponent. These changes in speed (*g* units, m/s^2^) are recorded using accelerometers fixed to the head, or protective equipment, such as helmets. Previous studies have established that linear accelerations of magnitudes of 66, 82 and 98 to 106 *g* are associated with 25, 50 and 80% risks of concussion, respectively [15,16]. Accelerometers also allow for measurement of head injury criterion (HIC), a score used to assess the probability of brain injury in both sports and motor vehicle contexts [17].

To date, most available literature on head impact emerges from studies performed on National Collegiate Athletic Association (NCAA) football players in the US. Results have shown that over the course of a single football season, it is estimated that players are susceptible to sustain as many as 1400 head impacts [18]. These studies have demonstrated that player position and type of plays are also highly associated with linear impact occurrence and magnitude [19] where for example, linemen are susceptible to fewer head impacts than skill players [19,20]. Despite available literature emerging from the US, knowledge remains limited about the potential application of these findings to Canadian football players. Indeed, American and Canadian football greatly differ due to game rules and these differences may potentially affect the applicability of current head impact literature. Differences in play, such as field size, scrimmage line, number of players on the field and length of playing season [21,22], could possibly affect the interpretation of short- and long-term head impacts risks. To our knowledge, no information about Canadian varsity football head impacts is currently available. Hence, the aim of this study was to characterize head impact exposure in Canadian varsity football players during regular season games. It is hypothesized that head impacts measures collected within the Canadian varsity system will differ from published data emerging from the USA.

## Methods

Football players from three varsity teams evolving in the Quebec University Football League in fall 2019 were enrolled in the study. Participants' height and weight and cumulative head impact index (CHII) were collected before the start of the football season. The CHII was developed based on head accelerometers studies and provides an estimate of head impacts received per football playing season from youth, high school and college years based on positions played [23]. Estimates of each seasons' are cumulated to provide a total score (CHII). It has previously been established that the CHII is a strong predictor of developing later-life cognitive impairments and that this risk increases unwaveringly every 1000 impacts [23]. Players were categorized into two groups – line players and skill players – based on the position they currently play. Participants gave informed consent at the beginning of the season and were assigned to data collection (i.e., wearing an instrumented head impact sensor) based on their injury status and traveling schedule (instrumentation was solely performed during local games in the greater Montreal area).

Participants' regular helmets were instrumented with the CUE sport sensor technology (Athlete Intelligence, WA, USA) for a maximum of four games per participant throughout the regular season. Helmets were adequately fitted by equipment managers of respective teams before the beginning of the season, and sensors were fixated in the helmet 2 h before each game, with double-sided glue, on the right side of the helmet, over the right ear opening. Sensors consist of a three-axis accelerometer coupled with a three-axis gyroscope, allowing to measure linear accelerations up to 200*g* [24]. Upon impact recording, telemetric values are sent by radio waves to the Athlete Intelligence platform, where information about individual impacts can be downloaded for further analysis at a later time. Magnitude, defined as linear acceleration (*g*) and HIC score were recorded by the CUE sport sensor. Solely impacts greater than 10*g* were recorded to avoid aberrant impacts due to movement of the head while running or jumping [19,25]. Games were recorded by two cameras from a lateral view with members of the research team recording entrances and exits from the playing field to cross-reference registered impacts. A total of 15 sensors were used across participants, and to ensure adequate filtration of relevant impacts (i.e. entrance/exit from field), three to six players were followed per game. This strategy to enhance the quality of our data collection unfortunately forced us to study small numbers of players.

## Analysis

Descriptive statistics with means and standard deviations were performed for all variables. Independent sample t-tests with significance set at p < 0.05 were performed to establish between-group differences for average magnitude, highest-recorded magnitude, cumulative force sustained within a game (addition of all impacts in one game per player), number of impacts, HIC score and highest HIC score. Given the high number of pairwise comparisons (six in total), a Bonferroni correction for multiple comparisons was applied, resulting in significance being set at p ≤ 0.008 for associated t-tests [26]. Statistical analyses were performed on IBM SPSS 26 statistical software.

## Results

Twenty-three varsity football players, aged between 20 and 24 years (mean: 23.25 ± 0.96), were followed for up to 4 games (mean: 2.26 ± 0.96; median: 2), resulting in sensor information for 56 events (i.e., players' head impacts per game were analyzed). Of the 23 players, six were linemen (three defensive linemen and three offensive linemen) and 17 were skill players (six linebackers, five defensive backs, four wide receivers and three running backs). Players were homogeneous in age but significantly differed (p < 0.005) in height and weight across playing positions (skill vs line players). Participants had been practicing football for an average of 12 seasons, with mean CHII score of 3259.18 ± 1389.80 and 4888.93 ± 810.00 for skill and line players respectively (refer to Table 1 for participants' characteristics). Due to the exploratory nature of this study and small sample size, differences across teams were not investigated.

**Table 1.** Characteristics of sample, with associated t-test and p-values.

	Total sample (n = 23)		Skill players^†^ (n = 17)		Linemen^‡^ (n = 6)		t	p-value
	M	SD	M	SD	M	SD		
Age (years)	23.26	0.96	23.15	1.05	23.53	0.62	-0.826	0.418
Height (cm)	183.54	6.51	181.24	5.31	190.08	5.18	-3.528	0.002
Weight (lbs)	219.39	41.27	199.47	20.70	275.83	30.82	-6.842	<0.001
Years playing football (all levels)	12.22	10.01	12.12	7.86	12.50	15.59	-0.079	0.938
Average playing time per game studied (min)	153.06	35.19	147.21	36.47	168.92	26.47	2.03	0.384
CHII score	3684.33	1445.44	3259.18	1389.80	4888.93	81000	-2.690	0.014

† Linebackers = 6; defensive backs = 5; wide receivers = 4; running backs = 3;

‡ Defensive linemen = 3; offensive linemen = 3.

CHII: Cumulative head impact index; M: Median; SD: Standard deviation; t: Independent sample t-test statistic.

**Figure 1.**
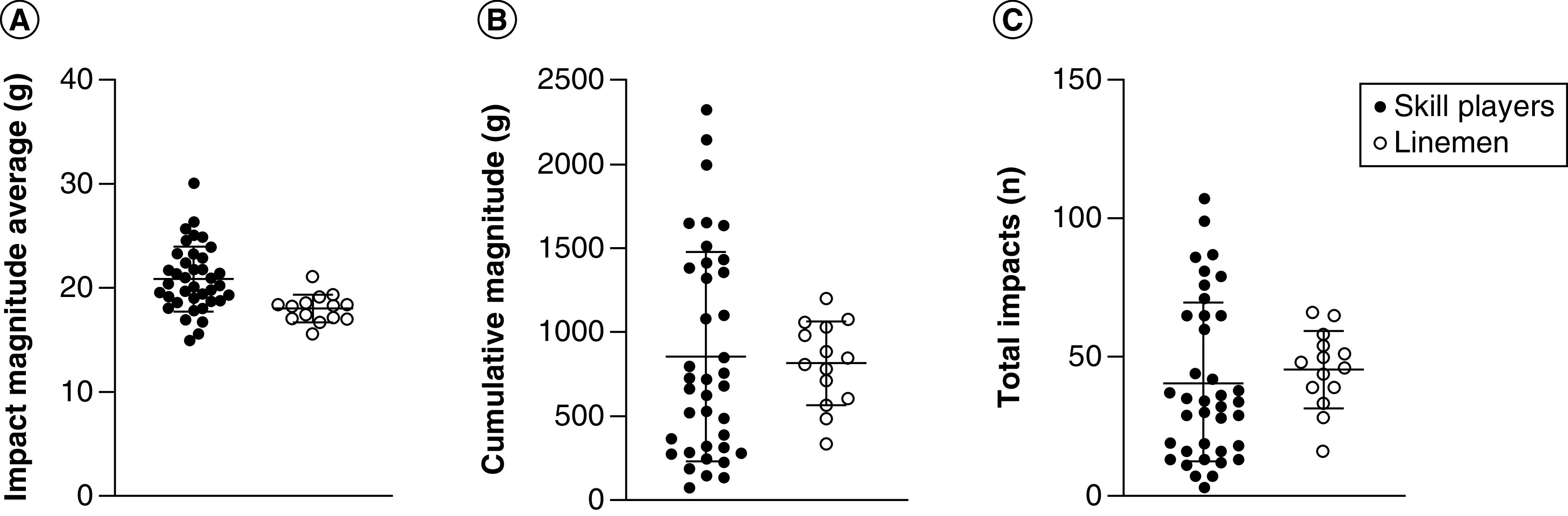
Magnitude and total amount of head impacts per playing position per game. **(A)** Impact magnitude average. **(B)** Cumulative magnitude. **(C)** Total number of impacts.

**Figure 2.**
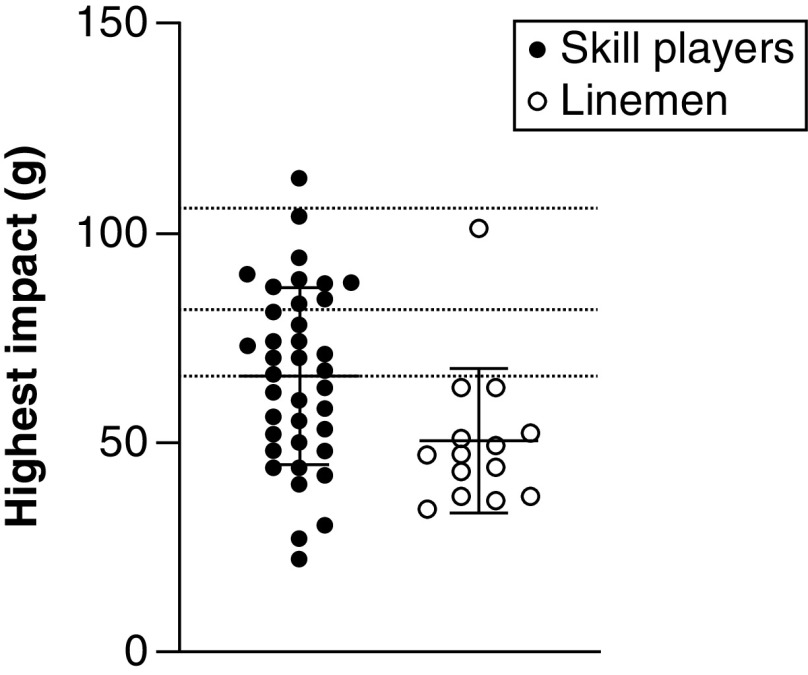
Highest impact per playing position within concussion threshold limits (66, 82 and 106 g).

**Figure 3.**
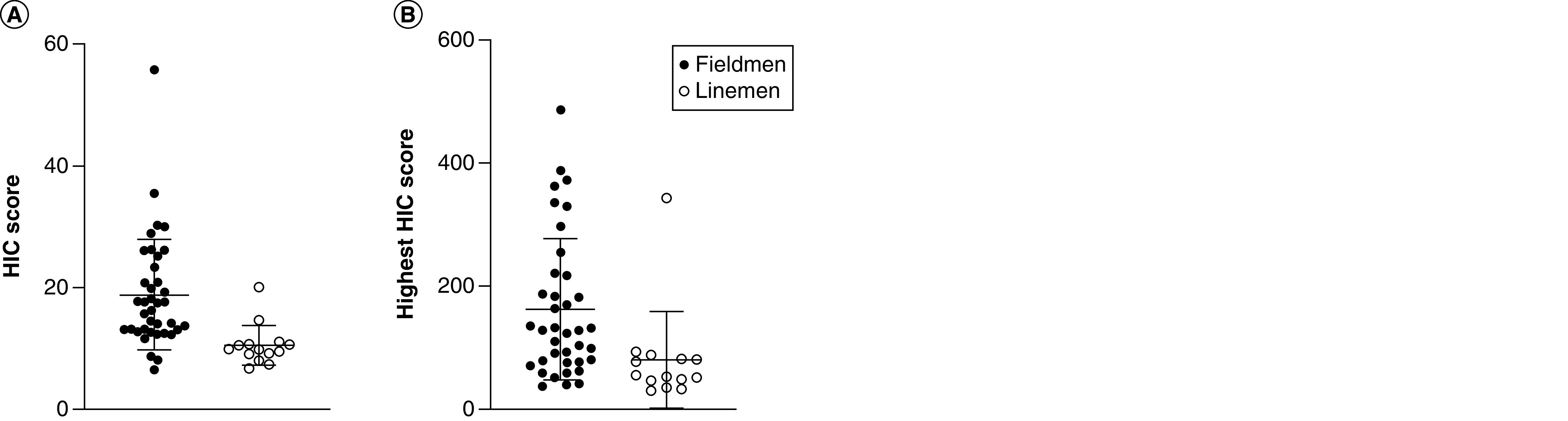
Head injury criterion score per playing position. **(A)** Average head injury criterion (HIC) score. **(B)** Highest recorded HIC score. HIC: Head injury criterion.

On average, in the course of a football game, players sustained 42.17 ± 25.55 hits (range: 3–107) to the head, with average magnitude of 20.08 ± 3.05 *g* and average HIC score of 16.64 ± 8.77 (refer to Figures 1 & 2). The highest magnitude recorded was of 113 *g* (average: 61.58 ± 21.25 g), and highest recorded HIC score was 486.76 (average: 139.83 ± 111.72). Cumulative impacts sustained within a game, when added for each player, were of 847.89 ± 545.21 *g* (range: 79–2326 g). Independent sample t-tests before Bonferroni correction revealed significant differences between groups in average impact magnitude (t = 4.554; p < 0.001; 95% CI: 1.59–4.10), highest recorded magnitude (t = 2.435; p < 0.02; 95% CI: 2.71–28.19), HIC score (t = 3.29; p = 0.029; 95% CI: 3.21–13.30) and maximal recorded HIC score (t = 2.925; p = 0.006; 95% CI: 25.08–139.29), with skills position players sustaining head impacts higher in magnitude and with higher HIC scores than linemen (Figure 3). After Bonferroni correction for multiple t-tests, the following comparisons remained significant: average impact magnitude (p < 0.001) and maximal recorded HIC score (p = 0.006). Between-group comparison of total number of impacts and cumulative impact sustained within a game did not reach significance (t = -0.764; p = 0.50; 95% CI: -16.55–7.44 and t = 0.376; p = 0.71; 95% CI: -197.79 –288.77, respectively). More than 98% of head impacts were below the 25% probability of concussion threshold previously established by the literature (Figure 2).

## Discussion

This exploratory study sought to portray head impacts sustained in the practice of American football in Canadian varsity athletes. Results of this study contribute to the existing body of literature in the field of head impact telemetry while providing preliminary insight on how this literature is applicable to the Canadian football population. A total of 2193 head impacts were analyzed from 23 athletes, for an average of 2 games per athlete, during the 2019 regular season. On average, players sustained 42.17 ± 25.55 impacts to the head during a game, with average magnitude of 20.08 ± 3.05 *g*, with increased amount of impacts and similar magnitude than studies coming from the US [18,25,27]. In this study, the vast majority of recorded head impacts were of low magnitude, with more than 98% below the 66 *g* (25% concussion-risk) threshold, similar to previously published studies in US football [18]. In 2010, Crisco *et al.* suggested that NCAA football players were susceptible of sustaining as many as 1444 head impacts per season [18]. Despite the Canadian football season involving one-third fewer games, results from our study suggest that Canadian football players would potentially be exposed to similar amounts of impacts through the course of the season. These results remain to be validated with a larger sample of football players. Surprisingly, CHII scores reported in our study were lower than those reported in the Montenigro *et al.* study, who estimated that college football players in the USA were susceptible to sustaining on average 6384.40 cumulative head impacts (CHII score) over an average of 12 years of playing football [23]. Our sample had similar football playing experience (12 years) with lower CHII score (3684.33). This raises questions on the applicability of CHII score with regard to Canadian football. Average HIC score was 16.64 ± 8.77, a value slightly below those reported by Duma *et al.*, with an average HIC score of 26 [28].

Overall, when comparing preliminary group values for head impacts in Canadian varsity football players, our results show that skill players are more likely to sustain impacts of higher magnitude and coincidently making them more prone to accumulate higher levels of cumulative impacts sustained with a game compared with linemen. Similarly, HIC scores were higher for skill players. Our results fall right within the lines of previously published work by researchers in the USA, where differences in head impacts were noted among playing positions [18–20,29,30].

To this day, it remains difficult to conclude on the applicability of NCAA football studies to the reality of Canadian football. Future studies with larger samples over the course of complete football seasons are necessary to contribute to the evidence of head impacts in the context of football. Although preliminary, results from this study are reliable due to the quality of the data collection that was coordinated with research assistants ensuring that recorded hits matched with time playing on the field, an issue often reported in this kind of experiment and known to cause false positives in recording hits [31]. Unfortunately, our study was unable to produce data on rotational accelerations, an important component to estimate brain damage [32]. Using sensors attached to helmets, deemed the analysis of such measures unreliable compared with other systems that are more elaborated and include gyroscopic systems, or that are closely fitted within the skull, such as instrumented mouth guards [24]. In addition, limited information is currently available on the validity and reliability of the CUE sport sensor, and validation studies are warranted before extrapolation of study results is possible. To gain a better understanding of the current head impact situation in Canada, recording of impacts during practices and through all regular season games would have helped shed light on similarities and differences of impacts during different venues over the span of a season. Although type of impacts (e.g., opponent, fall) were not reported in this particular study, such information would bring valuable insight to the literature. Such results could help in the long run to adapt measures of potential long-term sequelae due to repeated head impacts, such as the CHII, to the Canadian population. Finally, this study aimed solely to explore head impacts sustained to the head in the practice of Canadian varsity football regardless of concussion occurrence. Future studies should focus on the applicability of head impacts thresholds in the prediction of actual concussions sustained in the context of Canadian football.

## Conclusion

Despite this study involving a much smaller sample than usual studies on the matter, it has shown that it is feasible to study head impact kinematics in the Canadian football population and that preliminary results, in concordance with our hypothesis, suggest differences in head impact magnitude and occurrence when compared with studies from the USA. Although the practice of American football across the Canadian and US border may seem comparable, differences in head impact measures suggest that playing conditions may render the extrapolation of US data inadequate for Canadian athletes and warrant larger-scale studies on the north side of the border. Doing so could contribute to adapting readily available long-term sequelae prediction tools, such as the CHII, based on head impact occurrence to the Canadian population.

## Future perspective

The analysis of head impact kinematics should continue to evolve over the coming years in the hope to better understand the long-term effects of repetitive impacts to the head in the practice of sports. Future research in the field of head impact exposure specific to the Canadian population should include a broader age and level of play range, such as high school football. Such studies could encourage the comparison between Canadian and US football initiated in this work. Finally, research should also be encouraged in the context of other sports at risk of head impact exposure.

**Summary points**

- Canadian varsity football players sustain on average 42.17 ± 25.55 hits to the head during a game.
- Average head impact magnitude recorded is 20.08 ± 3.05 *g*.
- Canadian varsity football players sustain more hits than varsity football players in the USA, with similar impact magnitude.
- Consistent with studies from the National Collegiate Athletic Association, the vast majority of head impacts sustained in the practice of Canadian varsity football are below established concussion thresholds.
- Average head injury criterion score of individual head impacts are 16.64 ± 8.77.
- Skill players are more likely to sustain impacts of higher magnitude compared with linemen.
- Due to differences in playing contexts, it is unclear whether US prediction rules for long-term sequelae associated with football participation are applicable to Canadian university football athletes.
- Despite similar playing experience in years, cumulative head impact index scores greatly vary between Canadian and US athletes.
